# Biomarkers for Treatment Response in Orthodontics: Molecular Mechanisms, Clinical Utility, and Future Directions

**DOI:** 10.3390/ijms27125402

**Published:** 2026-06-16

**Authors:** Elzbieta Pawlowska, Maria Mitus-Kenig, Marcin Kozakiewicz, Janusz Blasiak

**Affiliations:** 1 Department of Developmental Dentistry, Medical University of Lodz, 92-215 Lodz, Poland; elzbieta.pawlowska@umed.lodz.pl; 2 Department of Periodontology, Preventive Dentistry and Oral Pathology, Faculty of Medicine, Jagiellonian University, 30-688 Krakow, Poland; maria.mitus-kenig@uj.edu.pl; 3 Department of Maxillofacial Surgery, Medical University of Lodz, 92-215 Lodz, Poland; 4 Faculty of Medicine, Mazovian University in Plock, 09-240 Plock, Poland

**Keywords:** orthodontics, biomarker, orthodontic tooth movement, epigenetic bone remodeling

## Abstract

Orthodontic tooth movement (OTM) is a biologically driven process resulting from the mechanically induced remodeling of the periodontal ligament (PDL) and alveolar bone. A marked inter-individual variability exists in the rate of tooth movement, susceptibility to adverse outcomes such as external apical root resorption (EARR), and overall treatment response. This narrative review synthesizes current evidence on molecular, genetic, and epigenetic biomarkers that underline these differences. We summarize established local biomarkers derived from gingival crevicular fluid and saliva, including inflammatory cytokines, matrix metalloproteinases, and bone remodeling mediators reflecting OTM compression- and tension-side biology. Beyond fluid biomarkers, growing attention is given to genetic and epigenetic determinants of OTM. Specific gene mutations are associated with impaired or absent tooth movement, while multiple single-nucleotide polymorphisms have been linked to increased risk of EARR. Recent studies further demonstrate that orthodontic forces induce epigenetic remodeling in PDL cells, including DNA methylation changes in the gene promoters, histone modifications, and force-responsive non-coding RNAs such as miR-21 and miR-146a, which collectively regulate osteoclastogenesis, inflammation, and tissue adaptation. These findings indicate that OTM is governed by an integrated network combining mechanical stimuli with genetic predisposition and dynamic epigenetic regulation. Understanding these mechanisms provides a foundation for the development of biomarker-guided, patient-specific therapeutic strategies.

## 1. Introduction

Orthodontic tooth movement (OTM), the key phenomenon in orthodontic treatment, is a biologically regulated process driven by a cascade of cellular and molecular events within the periodontal ligament (PDL) and alveolar bone [1]. Although the mechanical principles guiding orthodontic treatment are well-established, the biological response to orthodontic force varies widely among individuals [2]. Patients differ substantially in the rate of tooth movement, susceptibility to adverse effects such as external apical root resorption (EARR), periodontal inflammation, and treatment-related pain [3]. This variability reflects complex interactions between genetics/epigenetics, immune activity, bone remodeling capacity, systemic health, and local tissue microenvironment. Consequently, traditional biologically unstratified approaches to force selection and treatment planning are insufficient in the age of personalized medicine.

The idea of using biomarkers to predict orthodontic treatment response has gained considerable attention over the past decade [4]. Advances in molecular biology, genomics, imaging, and bioengineering have facilitated the discovery of various candidate biomarkers, including inflammatory cytokines, bone turnover markers, genetic polymorphisms, salivary and gingival crevicular fluid analytes, and imaging-based structural indicators. These biomarkers can predict treatment success, identify patients at risk of complications, and tailor bio-mechanical interventions based on individual biological profiles.

Research on orthodontic biomarkers has emerged in response to the need to better understand, measure, and predict the biological processes underlying OTM. While clinical orthodontics has traditionally relied on mechanical principles and observational outcomes, tooth movement is fundamentally a biologically regulated process that involves coordinated cellular-, molecular-, and tissue-level responses. Biomarker research aims to translate these biological events into measurable indicators that can guide diagnosis, timing, and treatment decisions. Its rationale is justified by several clinical and scientific motivations, which include (1) improving understanding of the biology of OTM; (2) enabling personalized and predictive orthodontics; (3) monitoring treatment progress in real time; (4) enhancing safety and minimizing adverse effects; (5) guiding accelerated orthodontic techniques, e.g., micro-osteoperforations, vibration, photobiomodulation; (6) supporting research on periodontally compromised patients; (7) integrating biology with digital and AI-driven orthodontics [5]. Therefore, the rationale for orthodontic biomarker research stems from the need to advance the specialty toward precision, personalization, predictability, and safety.

There are some, mainly systematic, reviews on the significance of biomarkers in orthodontics, e.g., refs. [6,7,8,9,10,11]. However, they remain largely descriptive and fragmented, with a predominant focus on cataloging individual molecules rather than interpreting their biological hierarchy, temporal dynamics, or clinical relevance. Most available syntheses concentrate on the initial phase of tooth movement, rely heavily on animal or in vitro data, and insufficiently address methodological heterogeneity in biomarker sampling, analysis, and interpretation. Moreover, clinically critical aspects, such as inter-individual biological variability, the prediction of adverse outcomes, biomarker behavior during finishing and retention phases, and translation into decision-making frameworks, have received limited and inconsistent attention. In parallel, recent advances in precision orthodontics and digitally guided treatment planning highlight the need for biologically informed models that integrate molecular markers with clinical workflows. Accordingly, a contemporary review is warranted to move beyond biomarker enumeration and provide an integrated, systems-based perspective that critically evaluates biological meaning, temporal dynamics, methodological limitations, and translational potential. Such a synthesis is essential for clarifying the current state of evidence, identifying knowledge gaps, and defining a coherent chain of actions needed to advance orthodontic biomarkers from experimental observations to clinically useful tools. Therefore, a thorough synthesis that provides and updates current information is needed to clarify the current state of research, assess the potential of specific biomarkers for translation into practice, and identify future research priorities. This review provides an integrated view by exploring biomarker categories, their underlying mechanisms, methodological issues, and clinical relevance. In doing so, it seeks to demonstrate how biomarker-based approaches could transform orthodontic practice and foster true precision orthodontics.

Our search strategy focused on the role of molecular, genetic, and epigenetic biomarkers and their correlations with imaging-derived markers in orthodontic diagnosis and treatment. It emphasized protein expression, DNA mutations, DNA methylation, histone modifications, non-coding RNA activity, RNA modifications, orthodontic forces, imaging, and tooth movement. It is based on available data regarding the mobility of both intact and endodontically treated teeth. The search drew on publications from PubMed, Embase, Google Scholar, ScienceDirect, and the Cochrane Library. We used search terms combining “orthodontic” with “biomarker,” “DNA,” “RNA,” “histone modification,” “miRNA,” “microRNA,” “siRNA,” “imaging,” or “RNA modification.” Publications from the past 10 years were prioritized unless earlier foundational studies were essential. The scope included human studies and relevant animal models. All article types, including original research, reviews, and meta-analyses, were included, with no language restrictions. We did not apply any parameters to select the most critical articles. Instead, we evaluated each based on essential content.

The aim of this work was to provide a comprehensive overview of the fundamental mechanisms underlying OTM, including the associated molecular events and key molecules involved. We further sought to critically evaluate various molecular markers, including biomarkers in gingival crevicular fluid, saliva, and serum. Particular emphasis was placed on genetic and epigenetic biomarkers. Additionally, imaging markers were discussed in relation to their interplay with molecular biomarkers. Finally, we examined both the established and potential clinical applications of these biomarkers, as well as the key challenges associated with their use.

## 2. Molecular Basis of Orthodontic Tooth Movement

**Figure 1:**
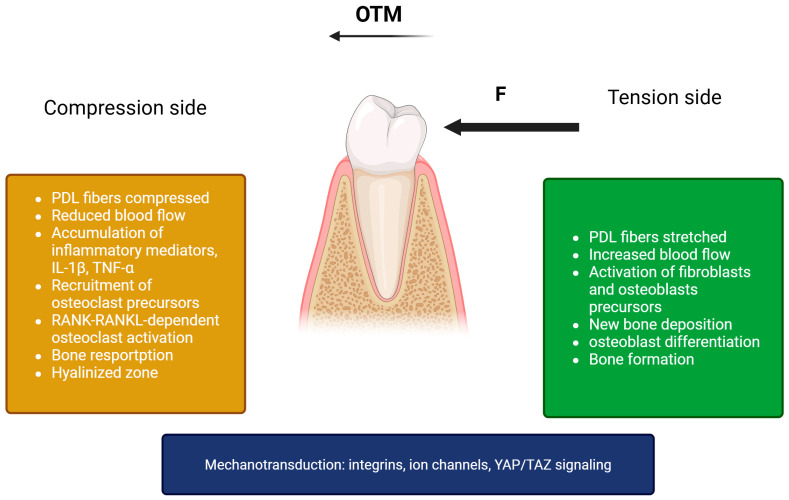
Bio-mechanical, cellular, and molecular processes underlying orthodontic tooth movement. Application of orthodontic force (F) creates distinct compression and tension zones within the periodontal ligament (PDL). On the compression side, mechanical deformation reduces vascular perfusion and induces the release of inflammatory mediators, including interleukin 1 beta (IL-1β) and tumor necrosis factor alpha (TNF-α), which promote the recruitment and differentiation of osteoclast precursors via the receptor activator of the nuclear factor κB (RANK)–RANK ligand (RANKL)–osteoprotegerin axis (RANK-RANKL) pathway. Osteoclastic resorption of alveolar bone creates space for tooth displacement. Transient hyalinized zones may form under heavy or sustained pressure and are removed by phagocytic cells. On the tension side, stretching of PDL fibers activates mechanotransduction pathways in fibroblasts and osteoblast precursors, enhancing osteoblastic bone deposition and stabilizing the tooth in its new position. Integrin-based signaling, ion channels, and transcriptional regulators such as yes-associated protein 1 and transcriptional co-activator with PDZ-binding motif (YAP/TAZ) coordinate these responses on both sides. Created in BioRender. Błasiak, J. (2026) https://BioRender.com/ie5soeo.

Orthodontic tooth movement is fundamentally a process of controlled, mechanically induced tissue remodeling within the PDL and surrounding alveolar bone. When an orthodontic force is applied to a tooth, it generates distinct regions of compression and tension in the PDL (Figure 1). These local bio-mechanical changes initiate a cascade of molecular and cellular events that ultimately allow the tooth to relocate within the alveolar socket [1].

In regions of compression, reduced blood flow and mechanical deformation stimulate the release of pro-inflammatory mediators, including cytokines, prostaglandins, and chemokines [12]. These signals recruit osteoclast precursors and promote differentiation into mature osteoclasts primarily through the receptor activator of nuclear factor κB (RANK)–RANK ligand (RANKL)–osteoprotegerin (OPG) axis. As osteoclasts resorb alveolar bone adjacent to the compressed PDL, space is created into which the tooth can move. The formation of transient hyalinized zones, areas of sterile necrosis, may temporarily slow movement until they are cleared by phagocytic cells [13]. Conversely, on the tension side, PDL fibroblasts, osteoblast precursors, and periodontal stem cells respond to stretching forces by upregulating pathways associated with bone formation. These include (1) canonical osteogenic pathway with wingless-related integration site proteins (WNTB)-catenin activation; (2) osteocyte regulation with parathyroid hormone-related protein-parathyroid hormone 1 receptor (PTHrP-PTH1R) leading to sclerostin suppression; (3) growth factor signaling, including bone morphogenetic proteins (BMPs), transforming growth factor beta (TGF-β), osteogenic signaling; (4) mechanosensory activation, including cilia and ion channels signaling, e.g., piezo; (5) tension-induced osteoblast differentiation [14,15,16,17,18]. Increased osteoblastic activity leads to the deposition of new bone, stabilizing the tooth in its updated position [15]. Mechanotransduction pathways involving integrins, ion channels, and transcriptional regulators such as yes-associated protein 1 (YAP), transcriptional co-activator with PDZ-binding motif (TAZ), and nuclear factor kappa-light-chain-enhancer of activated B cells (NF-κB) coordinate this remodeling process [19]. Mechanotransduction, specifically integrin signaling and the YAP/TAZ pathways, is the universal initial response to force in both tension and compression. The balance between resorption and formation reflects dynamic tissue remodeling and varies among individuals due to genetic, inflammatory, and systemic factors [20]. Figure 1 summarizes bio-mechanical, cellular, and molecular processes engaged in OTM on the compression and tension sides.

Therefore, OTM represents a dynamic equilibrium between bone resorption and bone formation, modulated by genetic and epigenetic background, systemic health, age, the local tissue microenvironment, and inflammatory responsiveness. This biological variability explains why patients differ in the rate of OTM and in susceptibility to treatment-related side effects. Understanding these processes provides the mechanistic basis for biomarker-based prediction and individualized orthodontic treatment planning.

## 3. Molecular and Biochemical Biomarkers of Orthodontic Therapy

### 3.1. Biomarkers in Gingival Crevicular Fluid

As an inflammatory exudate from the gingival vascular plexus, gingival crevicular fluid (GCF) undergoes characteristic compositional changes during the cellular remodeling of the PDL and alveolar bone triggered by orthodontic forces [6]. Therefore, GCF can be considered a minimally invasive tool for assessing bone turnover and tissue remodeling during OTM.

A wide range of inflammatory cytokines has been identified as sensitive GCF biomarkers during OTM [21]. Among these, IL-1B, IL-6, and TNF-α show consistent upregulation following the application of orthodontic forces, indicating early sterile inflammatory responses within the PDL. These mediators are among the most frequently reported differentially expressed proteins in proteomic analyses of GCF from orthodontic patients, supporting their role as reliable indicators of active tissue strain and osteoclastogenic signaling on the compression side of the moving tooth. Additionally, prostaglandin E2 (PGE2), a potent inflammatory mediator associated with osteoclast activation, consistently increases during the early phases of OTM, thereby further promoting bone resorption and tooth displacement [22].

Matrix degradation and remodeling are assessed by measuring matrix metalloproteinases (MMPs) in GCF. These proteolytic enzymes, especially MMP-1, MMP-3, MMP-8, and MMP-9, are often upregulated during OTM and indicate extracellular matrix turnover in the PDL under mechanical strain [23]. Integrated bioinformatic analyses have confirmed these MMPs as key components of the protein landscape involved in orthodontic tissue remodeling, suggesting their potential as markers for monitoring the rate and intensity of periodontal responses during treatment [21].

Central to the regulation of alveolar bone resorption is the RANKL/OPG axis, an important pathway in osteoclastogenesis. Studies show that RANKL levels increase and OPG levels decrease in GCF during OTM, particularly at compression sites, which correlates with a shift toward increased osteoclast recruitment and activation [24]. A randomized clinical trial assessing bone metabolism markers during early OTM showed significant changes in RANK and OPG expression at tension and compression sites, underscoring the diagnostic value of GCF for identifying spatially distinct molecular responses within the periodontium [25].

In addition to catabolic markers, GCF also contains anabolic indicators related to tension-side bone formation. Osteogenic molecules such as alkaline phosphatase (ALP) and TGF-β have been identified as potential players among the differentially expressed proteins during OTM [26,27]. Their upregulation corresponds to heightened osteoblastic activity and extracellular matrix deposition during alveolar bone remodeling on the tension side. Proteomic profiling also highlights ALP and TGF-β as two key bone formation-related markers present across the sequential bioprocesses that characterize OTM, suggesting their utility in tracking the transition from catabolic to anabolic remodeling phases [21].

A methodological limitation of GCF sampling is the risk of blood contamination, particularly in inflamed or mechanically irritated gingival tissues. Even minor contamination may introduce plasma-derived proteins and enzymes, leading to artificially elevated levels of inflammatory mediators and enzymatic activity, thereby confounding the interpretation of site-specific biomarker measurements.

In summary, GCF contains a wide range of molecular signatures, including cytokines, enzymes, structural proteins, and bone-regulatory factors, whose changes over time and in different locations closely follow the biological process of OTM. These biomarkers could be useful for developing diagnostic tools for the real-time monitoring of patient-specific responses to orthodontic forces. However, recent analyses highlight the difficulties arising from variability in GCF sampling, its contamination, measurement, and interpretation, underscoring the need for standardized protocols to validate biomarker utility across clinical settings [28].

### 3.2. Salivary Biomarkers

Saliva has gained increasing attention as an easily accessible, non-invasive diagnostic fluid that reflects the biochemical processes occurring during OTM, particularly bone remodeling dynamics and pain or stress responses [29]. Because mediators released during periodontal and alveolar bone turnover diffuse from the gingival crevice into whole saliva, salivary biomarkers offer a convenient, non-invasive alternative to GCF sampling for treatment monitoring [30]. Nevertheless, saliva is a diluted, systemically influenced medium and therefore lacks the site-specific precision of GCF, particularly for distinguishing localized biological responses at tension and compression sides around individual teeth. Saliva has been proposed as a non-invasive diagnostic medium reflecting both aspects of catabolic and anabolic phases of orthodontic tooth movement; however, most studies have predominantly focused on inflammatory and resorptive markers such as IL-1β, IL-6, RANKL, and MMPs [8]. To better capture the anabolic dimension of tissue remodeling, salivary biomarkers associated with bone formation, including ALP and growth factors, such as TGF-β, should also be considered [31]. Although these markers have been studied less extensively in saliva than in GCF, their inclusion may provide a more comprehensive representation of the dynamic balance between bone resorption and formation during orthodontic treatment. Additionally, salivary biomarkers of pain, stress, and neuroendocrine responses, including α-amylase, cortisol, secretory immunoglobulin A (IgA), and chromogranin A (CgA), have been associated with OTM [32]. Longitudinal clinical data show changes in these markers at the start of treatment, with salivary α-amylase showing a particularly strong response to orthodontic stress, although not all markers consistently align with self-reported pain levels [33].

Emerging research also emphasizes the diagnostic potential of salivary exosomal microRNAs (miRNAs) [34]. These molecules regulate gene expression involved in osteoclast and osteoblast differentiation, and differentially expressed miRNAs during OTM were identified, including hsa-miR-4634, which is implicated in bone remodeling pathways. This indicates a new class of biomarkers and highlights the importance of epigenetics in orthodontic therapy.

Taken together, current evidence indicates that saliva contains a wide range of measurable biomarkers reflecting inflammation, bone turnover, extracellular matrix remodeling, and pain-related physiology. While methodological challenges still exist, saliva remains a promising, non-invasive medium for tracking the biological progress of orthodontic treatment.

### 3.3. Serum Biomarkers

Although most molecular monitoring of OTM focuses on GCF or saliva, serum biomarkers have also been investigated as potential systemic indicators of bone remodeling triggered by orthodontic forces. Bone turnover markers in serum reflect whole-body skeletal metabolism and may provide insight into the systemic component of orthodontically induced bone turnover. A prospective clinical study evaluating bone remodeling markers in both GCF and serum during early stages of fixed appliance therapy examined two serum markers: C-terminal telopeptide of type I collagen (CTX), a marker of osteoclastic bone resorption, and procollagen type I N-terminal propeptide (PINP), a marker of osteoblastic bone formation [10]. The study found no significant changes in serum CTX or PINP levels over the first two weeks after force application, suggesting that early OTM may not produce measurable systemic alterations in bone turnover markers, or that any localized changes remain below the detection threshold in the blood due to dilution in the systemic circulation.

Despite the limited responsiveness of serum CTX and PINP to early orthodontic forces, serum biomarkers remain valuable for understanding patient-specific systemic bone metabolism during orthodontic therapy [35]. They can help identify inter-individual differences in baseline bone turnover: for example, serum CTX levels were higher in female participants, and serum PINP levels were higher in adults ≥25 years old, demonstrating that serum biomarkers may reflect age- and sex-dependent systemic bone physiology that could influence orthodontic responsiveness [10]. The systemic levels of TGF-β, OPG, and RANKL play essential roles in the regulation of bone formation and resorption [36].

While age- and sex-related variations in biomarkers such as PINP and CTX have been documented, a major limitation remains the lack of well-defined normative ranges across different age groups. This issue is especially critical in orthodontics, where biological responses differ substantially between growing individuals and adults due to variations in bone turnover rates and skeletal maturity. The absence of age-specific baseline values complicates the interpretation of biomarker measurements and represents a significant barrier to their integration into personalized treatment planning and clinical protocols.

Overall, serum biomarkers provide useful context for assessing global bone metabolism during orthodontic treatment, but their ability to reflect localized periodontal remodeling remains limited. Systemic dilution, short sampling windows, and relatively subtle local bone changes during OTM likely explain why serum markers have shown weaker diagnostic performance compared with GCF biomarkers [10]. As such, serum markers may complement, but not replace, local biological sampling when monitoring the molecular events associated with OTM.

## 4. Genetic and Epigenetic Biomarkers

Although mechanical forces initiate OTM, variation in treatment speed, tissue response, and risk of adverse outcomes is increasingly understood to be driven by genetic and epigenetic factors [37]. Recent studies have identified specific gene mutations, single-nucleotide polymorphisms (SNPs), and epigenetic modifications that influence the biology of PDL cells and the alveolar bone during OTM, offering opportunities for precision orthodontics [38].

### 4.1. Gene Mutations That Impair Orthodontic Tooth Movement

While most variability in OTM is polygenic, some monogenic conditions and gene mutations directly impair the biological ability for normal OTM [37]. One of the best-characterized impairments is the primary failure of eruption (PFE), which is caused by mutations in the parathyroid hormone receptor (*PTH1R*) gene [39]. Patients with *PTH1R*-related PFE show limited or no orthodontic movement because defective PTH signaling hinders bone remodeling and PDL responsiveness. Other Mendelian disorders, including those impacting the WNT signaling, osteoclast differentiation, collagen biosynthesis, and cementum formation, may also significantly impair or alter OTM mechanics [40]. These syndromes demonstrate how genetic impairments in osteoblast or osteoclast function can fundamentally change the skeletal response to orthodontic force.

### 4.2. Single-Nucleotide Polymorphisms Associated with Clinically Relevant Orthodontic Outcomes

Orthodontically induced inflammatory EARR is an unavoidable but undesirable pathological result of OTM [41]. EARR is the most extensively studied genetic trait in orthodontics, with multiple SNPs influencing individual risk.

The CG genotype of the rs1800587 polymorphism in the *IL1A* gene increased the risk of EARR in the Caucasian population, especially among individuals with increased overjet [42]. This work emphasizes the role of pro-inflammatory cytokine regulation in maintaining root integrity during OTM. The +3953 C > T polymorphism of the *IL1B* gene (rs1143634) was positively associated with the occurrence of EARR, confirming a key role for this cytokine in osteoclast recruitment and inflammatory remodeling under orthodontic force [43]. However, some studies question these observations, so further research is needed to clarify the role of this variability in the response to orthodontic treatment [44,45]. The –572 G > C SNP of the *IL6* gene (rs1800796) was linked to a higher risk of EARR [46]. This study showed that root resorption was greater in patients with the GC genotype than in those with the CC genotype. However, this research was limited to the central incisors. Variants of the IL-1 receptor antagonist (*IL1RN*) gene were linked to increased susceptibility to EARR, with stronger effects observed in female patients, indicating sex-specific inflammatory regulation [47]. Carriers of the G allele of the rs5275 polymorphism showed approximately 17% more mandibular molar root resorption, indicating prostaglandin metabolism’s role in root vulnerability during OTM [48]. A large 2024 cohort analysis identified links between the polymorphisms of actinin alpha 3 (*ACT3N*) and tuberous sclerosis complex 2 (*TSC2*) (rs678397 and rs1051771, respectively) and EARR, indicating that cytoskeletal organization and mechanosensitive signaling influence on how roots respond to compressive stress during movement [49].

Experimental work and phenotype–genotype correlations indicate that polymorphisms in genes that regulate bone remodeling, including RANKL, OPG, TGFB, and IL6, may affect the rate of tooth movement by shifting the balance between osteoclasts and osteoblasts. This is indirectly supported by SNP studies that show altered inflammatory and bone turnover responses during OTM, even when direct effects on movement rate were not measured [6].

### 4.3. Epigenetic Mechanisms Beyond Orthodontic Tooth Movement

The response of the PDL cells to mechanical stimuli is not determined solely by genetics, and epigenetic modifications can also play an important role in this process. High-resolution in vitro and ex vivo studies indicate that orthodontic forces cause rapid, reversible changes in DNA methylation, histone modifications, and non-coding RNA expression. Applying orthodontic force increases DNA methylation at the *RANKL* promoter, which reduces its expression while leaving *OPG* expression unchanged [4]. This epigenetically shifts the RANKL/OPG ratio, which controls osteoclast recruitment, thereby fine-tuning bone resorption on the compression side of the moving tooth. Therefore, DNA methylation of the RANKL gene acts as a molecular “brake” on osteoclast recruitment, reflecting a key epigenetic adjustment on the compression side of OTM. The same study observed that DNA methylation changes occur alongside histone activation marks, H3K27ac and H3K4me3, indicating an integrated epigenetic program in response to mechanical forces. Thus, this study demonstrates that DNA methylation is not an isolated event but part of a broader mechanotransduction–epigenetic response that shapes gene expression during OTM. It was also shown that DNA methylation regulates inflammatory genes in periodontal tissues and that altered DNA methylation patterns affect Toll-like receptor (TLR) pathways, cytokine expression, and osteoclastogenic signaling [50]. Since OTM is a sterile inflammatory process, the same methylation-regulated inflammatory pathways, including IL-1, TNF-α, and TLR signaling, are also activated during mechanical loading. This indirectly supports the involvement of DNA methylation in controlling OTM-induced inflammation.

A 2020 in vivo RNA-seq study identified 3075 differentially expressed genes (DEGs) during the first 14 days of OTM, with early genes linked to innate and adaptive immune activation and late genes associated with cytoskeletal remodeling, angiogenesis, and tissue homeostasis [51]. Although DNA methylation was not directly measured, the observed large-scale temporal reprogramming of gene expression is consistent with epigenetic regulation, particularly given the direct evidence of DNA methylation from the previously cited studies.

Experimental studies demonstrate that miRNAs integrate mechanotransduction signals with osteogenic and osteoclastic responses and help regulate inflammation, remodeling, and cellular adaptation to loading. A 2024 study that applied controlled compressive and tensile forces to human PDL fibroblasts demonstrated the upregulation of miR-21 under mechanical loading [52,53]. miR-21 expression increased most robustly after 48 h of force application. These studies have functional implications as miR-21 is known to promote osteoclast differentiation in other bone models, and its upregulation during OTM suggests a role in enhancing osteoclastic activity on the compression side, consistent with tooth movement biology. The same studies found that miR-146a exhibits a gradual, sustained increase in expression under both compressive and tensile loading. As miR-146a regulates innate immune signaling and acts as a negative feedback regulator of NF-κB-mediated inflammation, its mechanical upregulation suggests a role in modulating the sterile inflammatory response that characterizes early OTM. This places miR-146a at the intersection of mechanical stress, cytokine signaling, and the resolution of inflammation. Both miR-21 and miR-146a regulate gene networks essential for maintaining the balance between osteoblast and osteoclast activity during OTM.

Since tooth movement primarily relies on osteoclast-driven resorption on the compression side and osteoblast-driven formation on the tension side, and both cell types are heavily affected by epigenetic mechanisms, altering these pathways could potentially shift the remodeling balance to mimic the biological effects of force [54]. This hypothesis is supported by observations that demethylating the *RANKL* promoter enhances the osteoclastogenic response, inhibiting HDAC activity promotes osteoblast differentiation, and upregulating mechanosensitive miRNAs creates a “pseudo-mechanical” remodeling signal [55,56,57]. This led to the concept of “epigenetic tooth movement,” in which changes in cells involved in OTM might cause actual tooth displacement. A more practical alternative to force-free movement is that epigenetic interventions could enhance or accelerate OTM by increasing osteoclast recruitment, improving bone turnover, boosting responsiveness to mechanical forces, and helping resolve inflammation more quickly [58]. If future technologies allow precise delivery of epigenetic modulators to the PDL, biologically based orthodontic treatments could become possible. Significantly, this type of treatment could also be monitored and predicted using reliable molecular markers.

Single-cell RNA-sequencing of rat PDL under orthodontic force reveals that the ligament comprises 14 distinct cell clusters, each responding uniquely to mechanical loading [58]. After 1–2 weeks of force application, there were increases in collagen and extracellular matrix production, dynamic activation, and later resolution of sterile inflammation, as well as cluster-specific changes in mechanotransducers. Together, these findings reflect widespread, global shifts in PDL gene expression, not limited to a few isolated pathways, and provide a diverse array of molecular markers to monitor and predict the tooth response to orthodontic forces. Human RNA-seq studies have shown that orthodontic force triggers the coordinated activation of inflammatory, cell-cycle, and remodeling pathways [59]. Although the number of DEGs in this study is modest, the pathway-level activation suggests broad genome-wide responses affecting many cellular programs simultaneously.

### 4.4. Genetic–Epigenetic Interplay and Clinical Implications

The interaction between genetic predisposition and epigenetic modulation constitutes a complex regulatory network that governs bone remodeling during OTM [60]. Mechanical force initiates a cascade of intracellular events mediated by mechanosensitive receptors, cytoskeletal deformation, and extracellular matrix strain, ultimately leading to gene activation or silencing via epigenetic modifications. This interplay is essential for coordinating osteoclast and osteoblast activity during bone remodeling and contributes to differences in treatment rate, efficiency, and risk of adverse outcomes.

Table 1 presents genetic and epigenetic markers for monitoring OTM and predicting therapeutic outcomes. From a clinical standpoint, genetic and epigenetic biomarkers have significant potential to enhance precision orthodontics. Genetic markers may aid in identifying individuals predisposed to slower or faster tooth movement or a higher risk of root resorption [61]. Meanwhile, epigenetic biomarkers, because they are responsive to the application of force, may serve as dynamic indicators of ongoing remodeling, helping clinicians adjust treatment strategies in real time. Although translation into routine practice remains limited by the complexity of biomarker detection and the lack of standardized clinical protocols, the growing body of evidence supports the integration of genomics and epigenomics into future orthodontic diagnostics and personalized treatment planning [62].

Collectively, these findings demonstrate that OTM can be shaped by genomic background and mechanically induced epigenomic adaptation. Genetic polymorphisms, particularly in inflammatory and mechanosensitive pathways, help explain inter-patient differences in root resorption susceptibility and remodeling efficiency. Meanwhile, epigenetic modifications reflect real-time PDL responses and may serve as future dynamic biomarkers for monitoring tissue health and optimizing force application. As precision medicine advances, integrating genetic and epigenetic biomarkers into orthodontic diagnostics could predict movement rates, identify patients at risk for EARR, guide personalized force levels, and improve treatment safety and efficiency. Addressing clinical implementation will require standardized biomarker assays, larger longitudinal cohorts, and models that integrate genotype, epigenotype, and the mechanical environment.

## 5. Imaging-Derived Biomarkers

Imaging-derived biomarkers—quantitative, image-based metrics extracted from radiographic or 3D datasets—have become important for diagnostic precision, treatment planning, and the prediction of orthodontic outcomes [64]. Advances in 3D imaging, particularly cone-beam computed tomography (CBCT) and 3D facial surface scans, now allow clinicians to quantify anatomical and morphological changes that were previously invisible with 2D radiography. These imaging biomarkers are redefining the biological assessment of orthodontic treatment by enabling non-invasive, reproducible, and longitudinal evaluation of craniofacial structures [65].

CBCT-derived craniofacial metrics serve as imaging-derived structural correlates for evaluating skeletal relationships, airway dimensions, dental inclinations, alveolar bone morphology, and maxillofacial symmetry [66]. A 2024 CBCT-based analysis demonstrated the ability to extract metric and morphometric biomarkers from serial 3D datasets to quantify surgical or orthodontic modifications, including skull morphology, soft-tissue changes, and craniofacial adaptation [67]. These metrics allowed the objective comparison of pre- and post-treatment states, providing quantitative endpoints that function as imaging-derived biomarkers [67]. Such biomarkers offer several advantages in orthodontics as they (1) capture subtle skeletal and dental changes that influence treatment decisions; (2) support personalized force systems by providing detailed baseline anatomy; and (3) enhance predictability in complex cases by allowing simulation of treatment outcomes.

Bone and ligament remodeling in OTM occurs in well-defined temporal sequences and can be visualized radiographically as structural biomarkers [17]. These include changes in trabecular density, bone deposition and resorption patterns, and root surface resorption, all of which can be measured using advanced imaging techniques. Incorporating these imaging biomarkers may help clinicians identify early signs of root resorption and adjust forces accordingly. Additionally, these markers can support the monitoring of alveolar bone adaptation to maintain periodontal health and the selection of an optimal force level to prevent iatrogenic damage [68].

As OTM is fundamentally a biologically mediated process, imaging biomarkers provide a visual correlate to underlying molecular and cellular pathways such as sterile inflammation, osteoclastic resorption, and osteoblastic bone formation [69]. Periodontal remodeling studies demonstrate that structural changes seen on CBCT mirror biological phenomena such as PDL cell strain and remodeling, osteoclastic lacunae formation, angiogenesis, and changes in vascular spaces and cortical modeling on the tension and compression sides [70,71]. The ability to align these imaging-derived biomarkers with known bio-mechanical and molecular processes advances the field toward biologically informed orthodontics [17].

## 6. Biomarkers Related to Orthodontic Pain and Neurogenic Response

Orthodontic pain arises primarily from the inflammatory and neurogenic responses triggered by mechanical forces applied to teeth. These forces initiate cellular, vascular, and neural events within PDL, GCF, and surrounding bone, producing measurable biochemical markers that can serve as objective indicators of tissue response and pain perception. Mechanical strain activates periodontal nociceptors, particularly those expressing transient receptor potential vanilloid 1 (TRPV1), transient receptor potential ankyrin 1 (TRPA1), acid-sensing ion channel subunit 3 (ASIC3), and purinergic receptor P2X, which transduce noxious stimuli and contribute to pain signaling pathways [71]. These nociceptors also modulate inflammatory cascades and bone remodeling. Increased activity of calcitonin gene-related peptide (CGRP) and substance P (SP), key molecules in migraine-related pain, is consistently associated with heightened neurogenic inflammation and orthodontic discomfort, making them key biomarkers of neural response to orthodontic force [72,73]. It was demonstrated that the CGRP and SP expression increased during OTM, reflecting the activation of sensory neurons [74]. It was also shown that sensory nerve injury suppresses OTM-induced increases in CGRP immunoreactivity, confirming an integral role of CGRP in the neurosensory response and bone turnover coupling [75]. Brain-derived neurotrophic factor (BDNF) has emerged as an additional neurogenic biomarker. Clinical studies demonstrated that salivary BDNF levels, an essential factor in neuronal survival and growth, correlated with subjective pain intensity during initial orthodontic activation and outperformed CGRP in this regard [76].

The PDL environment responds rapidly to orthodontic force with a surge in pro-inflammatory cytokines, including IL-1β, IL-6, IL-8, TNF-α, which contribute to pain and initiate bone resorption at compression sites [77]. Early peaks in IL-Iβ were directly associated with the rate of tooth movement and inflammatory sensitization [78]. Systematic evaluations show that orthodontic appliances elevate these cytokines in GCF shortly after force application, confirming their utility as biomarkers of both inflammation and discomfort [79]. The interplay of anti-inflammatory cytokines, such as IL-10, IL-4, and IL-1RA, follows in later phases, corresponding to the repair and attenuation of pain. Cytokines also function as key mediators of immunoinflammatory responses in bone remodeling, linking mechanical stimuli, pain, and osteoclastic/osteoblastic activity [80].

Studies of GCF show notable increases in substance P levels in teeth with pain compared to healthy controls, supporting SP as a reliable biomarker of dental pain and neurogenic inflammation [81,82]. Similarly, saliva-detectable biomarkers, including α-amylase (sAA), cortisol, secretory immunoglobulin A (sIgA), chromogranin A, and neuropeptides, reflect sympathetic and stress-related contributions to orthodontic discomfort. However, longitudinal studies note that sAA spikes are more strongly associated with treatment-related stress than with tooth-movement pain per se [33]. Biomarkers such as osteocalcin, MMPs, and components of the RANK/RANKL/OPG system are integral to bone turnover and indirectly relate to pain by shaping tissue inflammation and remodeling rates [83,84,85,86]. Neurogenic mediators, in particular CGRP, can enhance osteoclastic activity, thereby demonstrating a direct functional link between neural activation and the biology of mechanical tooth movement [87].

Recent studies emphasize the complex interplay among sensory neurons, glial activation, oxidative stress, and epigenetic regulation in shaping orofacial pain, indicating that biomarkers of orthodontic pain must be understood within a broader neuroimmune context [88]. In addition, nociceptive molecules such as TRPV1 not only mediate pain but also influence alveolar bone remodeling, representing a promising frontier for therapeutic interventions that reduce pain while accelerating tooth movement [71].

The principal molecular biomarkers investigated in orthodontics can be grouped according to their biological function, anatomical origin, and potential clinical application, as summarized in Table 2.

## 7. Clinical Translation and Practical Application

The increasing understanding of molecular events underlying OTM has created opportunities to translate biomarker research into clinical diagnostics, treatment optimization, and personalized orthodontic care. Human studies have demonstrated that osteocalcin levels in GCF change during orthodontic treatment, including periodontally compromised teeth undergoing canine retraction [89,90]. Although the changes were modest, they reflected biological remodeling over months, indicating potential for monitoring bone turnover in clinical settings. Osteocalcin’s responsiveness to mechanical load makes it a promising marker for identifying periods of active bone formation or resorption, delayed or insufficient biological response, and patient-specific variability in tissue adaptation [91].

MMP-1 and MMP-2 concentrations in human GCF rise within hours after application of orthodontic force, reflecting early ECM degradation and PDL remodeling [83]. In practice, these MMP fluctuations could support (1) the verification of adequate force application and PDL response; (2) the early detection of adverse tissue reactions or excessive inflammatory load; (3) timing adjustments for follow-up activations to match biological readiness.

A longitudinal human salivary study showed increases in soluble RANKL, decreases in OPG, and a rising RANKL/OPG ratio during orthodontic treatment phases [92]. Since the RANKL/OPG balance governs osteoclast activity, monitoring it clinically may help (1) identify individuals prone to rapid bone resorption; (2) predict susceptibility to root resorption; (3) tailor force levels to minimize unwanted tissue damage.

Biomarkers enable clinicians to distinguish biologically fast vs. slow responders. For example, patients with early spikes in MMPs or RANKL may require lighter forces or longer intervals, but those showing blunted biochemical responses might benefit from adjunctive acceleration therapies [93,94].

Studies analyzing osteocalcin levels in adults with periodontal involvement have shown stable yet measurable trends over months of orthodontic movement [90]. This suggests that biomarkers can help ensure that orthodontic forces remain within a biologically safe range and provide a method for monitoring tissue stability during prolonged treatment. Patients with reduced bone density or inflammation-altered metabolism could benefit from the biomarker-guided fine-tuning of force, modification of activation schedules, prediction of treatment rate, and stability [95,96].

Adjunctive procedures such as micro-osteoperforations, corticotomies, and vibration devices aim to exploit the Regional Acceleratory Phenomenon (RAP), a temporary increase in bone and soft-tissue remodeling activity in a specific area following trauma [97,98]. Biomarker data can serve as objective verification tools. For instance, increased osteocalcin and IL1 levels after micro-osteoperforation provide measurable evidence of enhanced turnover in human GCF [99]. This supports clinical decisions regarding the optimal timing of activation intervals, the evaluation of the efficacy of acceleration procedures, and the minimization of over-treatment or unnecessary interventions.

Given that biomarkers such as MMPs, osteocalcin, and sRANKL/OPG are detectable via minimally invasive GCF or saliva sampling, future developments may include rapid chairside immunoassay kits for orthodontists, real-time “biological readiness” profiling prior to activations, and biomarker-driven decision algorithms integrated into digital orthodontic workflows.

From a clinical perspective, orthodontic biomarkers offer several practical applications that extend beyond experimental observation. In routine practice, biomarkers detected in GCF or saliva may support the assessment of inflammatory status, tissue remodeling activity, and individual biological responsiveness to orthodontic forces. Their incorporation into the clinical process can be conceptualized across treatment stages, including baseline evaluation, the monitoring of force-induced responses during activation visits, and the assessment of stabilization during the finishing and retention phases. Such integration may complement traditional bio-mechanical planning by introducing a biological dimension to treatment decisions, including adjustments to force magnitude and activation timing, and identifying patients at increased risk of adverse effects, such as excessive inflammation or root resorption.

Orthodontic biomarkers have transitioned from purely experimental tools to emerging clinical companions that guide treatment timing, force magnitude, risk assessment, and patient-specific biological adaptation. Continued refinement of sampling protocols and rapid-testing technologies will likely make biomarker-driven orthodontics an integral part of daily practice. Despite the promising potential of orthodontic biomarkers, several practical and methodological challenges must be addressed before routine clinical implementation is feasible. These include concerns regarding the cost-effectiveness of repeated molecular testing, the need for standardized sampling and analytical protocols, and substantial inter-individual variability in biomarker expression. In addition, access to specialized laboratory or chairside molecular diagnostic facilities remains limited, and ethical considerations regarding patient stratification and data interpretation must be addressed. Crucially, most biomarkers lack robust longitudinal validation and clearly defined clinical thresholds, underscoring the need for further high-quality human studies before they can be reliably integrated into clinical practice.

Looking ahead, the clinical translation of orthodontic biomarkers will likely depend on standardized protocols, validated biomarker panels, and accessible diagnostic technologies. Advances in chairside testing, microfluidic platforms, and digital and AI-supported orthodontics may enable the real-time assessment of biological responses, facilitating a shift toward biologically guided, personalized treatment strategies. In particular, integrating biomarker data with AI and predictive modeling offers a promising avenue for optimizing treatment efficiency and patient-specific outcomes. However, achieving this level of clinical application will require robust longitudinal validation and clear evidence of cost-effectiveness and added clinical value.

## 8. Conclusions, Knowledge Gaps, Outstanding Questions, and Perspectives

Biomarker-driven approaches may ultimately allow orthodontists to forecast tooth movement rates, anticipate adverse effects, personalize force systems, and improve patient comfort and safety [77]. By uniting mechanistic biology with clinical orthodontics, biomarker science stands poised to enable precision orthodontics, transforming both research frameworks and everyday practice.

Despite advances in understanding molecular and cellular responses associated with OTM, substantial knowledge gaps remain, limiting the clinical translation of biomarker-based diagnostics. The current literature highlights shortcomings in human data, longitudinal characterization, biomarker interactions, predictive modeling, and the integration with emerging digital technologies [4,5,6,8,100,101].

Many studies in orthodontic biology are conducted using animal models or in vitro systems, whereas rigorously designed human studies remain scarce. Furthermore, heterogeneous patient populations and small sample sizes impede efforts to define normative biomarker ranges across ages, periodontal conditions, and biological phenotypes [5]. Therefore, it is important to determine what constitutes normal versus pathological variation in human biomarkers and how age, sex, genetics, and periodontal health affect biomarker expression.

The methodology for assessing GCF and saliva biomarkers lacks uniformity. Studies differ markedly in sampling sites, with timing relative to force application, volume collection techniques, storage conditions, and analytical assays. Such inconsistency leads to poor reproducibility and hinders cross-study comparisons, thereby preventing the development of unified clinical guidelines [102].

Another problem is a limited understanding across all treatment phases. Most biomarker studies focus on the initial phase of OTM, whereas the mid-treatment, finishing, and especially retention/relapse phases remain underexplored [4]. It is striking that the retention period, during which relapse risk is highest, has attracted little biochemical investigation. Therefore, it is important to address two questions: (1) How do biomarker profiles evolve longitudinally across full treatment cycles? (2) Which molecular signatures predict relapse or long-term stability?

Patients exhibit markedly different biological responses to similar orthodontic forces [2]. Therefore, it is justified to explore whether biomarker screening can categorize patients into “fast,” “average,” and “slow” responders, and which genetic/epigenetic, or systemic factors influence biomarker responsiveness.

Although numerous markers correlate with tissue remodeling, no clinically validated predictive models currently exist to guide force magnitude, activation intervals, or treatment duration. Although current reviews forecast increasing integration of biomarkers into predictive modeling, they note that the field lacks validated algorithms capable of real-time decision support [103]. Further studies should determine how many biomarkers, and which combination, would yield clinically reliable predictions. Also, can machine learning incorporate longitudinal biological data to optimize treatment and what biomarker types are most suitable for integration into AI-driven prediction models? Another problem in this context is the individualization of biomarkers for patients with compromised periodontal support.

In summary, orthodontic biomarker research has evolved from descriptive molecular profiling to a more integrative, clinically oriented discipline, yet its full translational potential remains unrealized. Bridging this gap will require not only methodological rigor and robust human validation but also a conceptual shift toward the systems-based interpretation of biological signals in the clinical context. Integrating standardized, longitudinal, and multimodal biomarker data alongside advances in digital technologies and predictive analytics offers a realistic pathway toward biologically guided treatment strategies. Ultimately, the successful implementation of biomarkers in orthodontics will depend on their ability to enhance clinical decision-making, improve patient-specific outcomes, and align mechanistic insights with practical applicability, thereby advancing the field toward true precision orthodontics.

## Figures and Tables

**Table 1:** Genetic and epigenetic biomarkers relevant to bone remodeling, orthodontic tooth movement (OTM), and external apical root resorption (EARR).

Category	Biomarker/Gene	Variant/Mechanism	Effect on OTM and Clinical Relevance	Reference ^*a*^
Genetic markers associated with OTM, EARR, and bone remodeling	*PTH1R ^b^*	Loss-of-function mutations	PFE → no OTM	[8,9]
	WNT-pathway genes	Pathogenic mutations	Altered bone modeling → reduced or distorted OTM	[8,9]
	Skeletal dysplasia-related genes	Mutations affecting osteoclast/osteoblast biology	Impaired remodeling → reduced or abnormal OTM	[9]
	*IL1A*	rs1800587 (GG genotype)	Predisposes to EARR	[63]
	*IL1B*	Specific SNP	Increases odds of EARR fourfold	[6]
	*IL1RN*	VNTR variants	Associated with EARR, especially in females	[36]
	*COX2*	rs5275 (G allele)	17% increase in EARR	[48]
	*ACT3N*	rs678397	EARR association	[5]
	*TSC2*	rs1051771	EARR association	[5]
	RANKL/OPG pathway genes	Polymorphisms	Osteoclast/osteoblast balance	[6]
	Cytokine genes, e.g., *IL6*	Inflammatory SNPs	Alter inflammatory response	[6]
Epigenetic Modifications	*RANKL* promoter	Increased DNA methylation	Reduces RANKL expression → modulates osteoclastogenesis	[4]
	Global DNA changes	Force-dependent methylation shifts	Alters PDL transcriptional landscape	[4]
	H3K27ac	Upregulated under force	Promotes the transcription of remodeling genes	[4]
	H3K4me3	Upregulated under force	Osteogenic gene activation	[4]
	miR-21	Upregulated	Promotes osteoclastogenesis and inflammation	[4]
	miR-146a	Upregulated	Modulates immune mechanotransduction	[4]
Global gene expression	>3000 DEGs	Multiple pathways activated	Early immune activation; later proliferation and angiogenesis	[9]

*^a^* To facilitate a complex analysis, review papers were referenced. *^b^* Acronyms: ACTN3, actinin alpha 3; COX2, cyclooxygenase 2; DEG, differentially expressed gene; EARR, external apical root resorption; H3K27ac, histone H3 lysine 27 acetylation; H3K4me3, histone H3 lysine 4 trimethylation; IL1B, interleukin-2 beta; IL1RN, interleukin-1 receptor antagonist; IL6, interleukin-6; OPG, osteoprotegerin; PFE, primary failure of eruption; PHR1, parathyroid hormone 1 receptor; RANKL, receptor activator of nuclear factor-κB ligand; SNP, single-nucleotide polymorphism; TSC2, tuberous sclerosis complex 2; VNTR, variable number of tandem repeat; WNT, wingless-related integration site.

**Table 2:** Key molecular biomarkers in orthodontics across biological systems and clinical relevance according to the current consensus in the field. The genetic and epigenetic markers are presented in Table 1.

Biomarker Class	Anatomical Origin	Proposed Application	Level of Evidence	Current Challenges
Pro-inflammatory cytokines (IL-1β *^a^*, TNF-α, IL-6, IL-8, IL-2, IFN-γ)	PDL, GCF, saliva	Early detection of inflammatory response to orthodontic forces; monitoring tissue activation; indirect correlation with pain	Moderate–high (human GCF studies + animal models)	High inter-individual variability; lack of standardized sampling timing; transient expression peaks
Anti-inflammatory cytokines (IL-10, IL-4, IL-1RA)	GCF, PDL, immune cells	Monitoring resolution phase of inflammation; evaluation of tissue recovery and healing	Low–moderate	Limited longitudinal studies; unclear clinical thresholds; underexplored in orthodontics
Bone remodeling markers (RANK, RANKL, OPG, cathepsin K)	Osteocytes, osteoblasts, PDL; detectable in GCF and saliva	Assessment of osteoclast activation; prediction of bone resorption rate; potential risk indicator for root resorption	Moderate (human + strong animal evidence)	Lack of defined clinical cut-off values; variability between sampling media (GCF vs. saliva)
Bone formation markers (ALP, OC, type-I collagen, RUNX2)	Osteoblasts, alveolar bone; detectable in GCF and serum	Monitoring osteoblastic activity; evaluation of bone formation phase; assessment of treatment progression	Moderate (human clinical studies available)	Small effect sizes in humans; slow temporal changes limit real-time application
Matrix metalloproteinases (MMP-1, MMP-2, MMP-8, MMP-9)	PDL fibroblasts, inflammatory cells; GCF	Detection of extracellular matrix remodeling; early indicator of force response; potential marker of tissue breakdown	Moderate–high (human studies with short-term measurements)	Rapid fluctuations; influenced by periodontal status; lack of specificity to orthodontic processes
Neuropeptides (substance P, CGRP, BDNF, VIP)	Sensory nerve endings in PDL and pulp; saliva and GCF	Assessment of neurogenic inflammation; correlation with orthodontic pain perception	Moderate (human and animal studies)	Difficult standardization; strong influence of psychological and systemic factors
Stress-related salivary markers (cortisol, aAA, cGA)	Salivary glands (systemic origin)	Evaluation of stress response to orthodontic treatment; adjunct in pain assessment	Low–moderate	Low specificity for orthodontics; influenced by circadian rhythm and external stressors
Prostaglandins (PGE2)	PDL cells, inflammatory cells; GCF	Marker of inflammatory activation and bone resorption; mediator of tooth movement rate	Moderate (human + animal evidence)	Very short half-life; difficult to measure reliably; invasive sampling timing critical
Growth factors (TGF-β, BMP, FGF-2, PDGF)	Osteoblasts, endothelial cells, PDL	Assessment of angiogenesis and tissue remodeling; possible marker of healing and adaptation	Low–moderate	Limited orthodontic-specific human data; complex interaction with other pathways
Mechanotransduction-related markers (integrins, FAK, TRPV1, TRPA1)	PDL cells, sensory neurons	Understanding force sensing and initiation of biological response; potential targets for personalized force application	Low (mostly experimental/animal data)	Lack of clinical validation; not yet measurable in routine clinical fluids

*^a^* Acronyms: PDL, periodontal ligament; GCF, gingival crevicular fluid; IL-1β, interleukin 1β; TNF-α, tumor necrosis factor-alpha; RANKl, receptor activator of nuclear factor-κB ligand; IFN-γ, interferon gamma; RUNX2, Runt-Related Transcription Factor 2; MMP, matrix metalloproteinase; ALP, alkaline phosphatase; OC, osteocalcin; OPN, osteopontin; CGRP, calcitonin gene-related peptide; BDNF, brain-derived neurotrophic factor; VIP, vasoactive intestinal peptide; sAA, salivary alpha-amylase; cGA, chromogranin; TGF-β, transforming growth factor beta; PGE2, prostaglndin E2; BMP, bone morphogenetic protein; PDGF, platelet-derived growth factor; FAK, focal adhesion kinase; TRPV1, transient receptor potential vanilloid 1; TRPA1, transient receptor potential ankyrin 1.

## Data Availability

No new data were created or analyzed in this study. Data sharing is not applicable to this article.
